# The induction and identification of novel Colistin resistance mutations in *Acinetobacter baumannii* and their implications

**DOI:** 10.1038/srep28291

**Published:** 2016-06-22

**Authors:** Nguyen Thi Khanh Nhu, David W. Riordan, Tran Do Hoang Nhu, Duy Pham Thanh, Guy Thwaites, Nguyen Phu Huong Lan, Brendan W. Wren, Stephen Baker, Richard A Stabler

**Affiliations:** 1 The Hospital for Tropical Diseases, Wellcome Trust Major Overseas Programme, Oxford University Clinical Research Unit, Ho Chi Minh City, Vietnam; 2 School of Chemistry and Molecular Biosciences, The University of Queensland, Brisbane, Queensland, Australia; 3 Department of Pathogen and Molecular Biology, London School of Hygiene & Tropical Medicine, London; 4 Centre for Tropical Medicine, Nuffield Department of Clinical Medicine, Oxford University, United Kingdom; 5 The Hospital for Tropical Diseases, Ho Chi Minh City, Vietnam

## Abstract

*Acinetobacter baumannii* is a significant cause of opportunistic hospital acquired infection and has been identified as an important emerging infection due to its high levels of antimicrobial resistance. Multidrug resistant *A. baumannii* has risen rapidly in Vietnam, where colistin is becoming the drug of last resort for many infections. In this study we generated spontaneous colistin resistant progeny (up to >256 μg/μl) from four colistin susceptible Vietnamese isolates and one susceptible reference strain (MIC <1.5 μg/μl). Whole genome sequencing was used to identify single nucleotide mutations that could be attributed to the reduced colistin susceptibility. We identified six *lpxACD* and three *pmrB* mutations, the majority of which were novel. In addition, we identified further mutations in six *A. baumannii* genes (*vacJ*, *pldA*, *ttg2C*, *pheS* and conserved hypothetical protein) that we hypothesise have a role in reduced colistin susceptibility. This study has identified additional mutations that may be associated with colistin resistance through novel resistance mechanisms. Our work further demonstrates how rapidly *A. baumannii* can generate resistance to a last resort antimicrobial and highlights the need for improved surveillance to identified *A. baumannii* with an extensive drug resistance profile.

*Acinetobacter baumannii* is emerging worldwide as a hospital acquired pathogen infecting critically ill patients. In susceptible individuals *A. baumannii* can cause a range of infections including pneumonia, bacteraemia, meningitis, blood stream infections and urinary tract infections^1^,^2^. On rare occasions *A. baumannii* has been linked to infective endocarditis^3^,^4^. *A. baumannii* can be a serious complication of serious trauma including cellulitis and soft-tissue infection^5^ as exemplified in military personnel returning from Iraq^6^ and Vietnam^7^. In addition, it is currently estimated to be responsible for 1 in 5 cases of ventilator-associated pneumonia (VAP) in Europe^8^.

The rapid emergence of *A. baumannii* has been associated with increasing resistance against the majority of currently used antimicrobials. In our previous study, conducted in an ICU in southern Vietnam over an 11-year period (2000–2011), we found that *A. baumannii* had become the principle pathogen causing VAP; this increase in prevalence was concurrent with the emergence and maintenance of resistance to carbapenems^9^. The ability of *A. baumannii* to develop broad spectrum resistance to antimicrobials through intrinsic and acquired antimicrobial resistance means that treating persistent *A. baumannii* infections is a significant clinical challenge^10^. Resistance to carbapenems, such as meropenem and imepenem, which were considered to be the “gold standard” for the treatment of *A. baumannii* infections^11^,^12^, has resulted in colistin, a member of the polymyxin group of antimicrobials, becoming the last treatment option. Colistin is, therefore, a key drug in treating and controlling many hospital acquired infections due to multiresistant Gram-negative bacteria.

The action of colistin is similar to that of a detergent. The outer membrane of the bacterial cell is disrupted through electrostatic binding of positively charged regions of the colistin and negatively charged regions of the hydrophilic lipid A component of lipopolysaccharide (LPS)^13^. This disruption of the lipid A component of the LPS induces a loss of integrity in the cell membrane and ultimately cell death. However, a reduction in the net negative charge of the LPS through modifications in lipid A decreases the binding potential of colistin to bacterial membrane, which leads to reduced susceptibility against colistin^14^. The first genetic mechanism of colistin resistance to be reported was the *pmrAB* two-component system^15^. In *Salmonella*, PmrAB alters expression of the lipid A modification genes in response to environmental pH, Fe3 + and Mg2 + levels^16^,^17^. PmrA-regulated resistance in *Salmonella* results in the modification of lipid A with either 4-deoxy-aminoarabinose (Ara4N) or phosphoethanolamine. However, *A. baumannii* lacks the Ara4N biosynthesis and/or attachment genes. Laboratory induced colistin resistance in *A. baumannii* has been demonstrated to be through non-synonymous mutations in both *pmrA* or *pmrB,* but not *pmrC*^15^,^18^. Adams *et al.* also showed that these mutations were clinically relevant through identification of a non-synonymous mutation in *pmrB* in clinical *A. baumannii* isolates at the same residue as in one of the laboratory induced isolates^15^. PmrA is responsible for auto-regulation of the operon and it has also been demonstrated that PmrA up-regulation is required for resistance^14^,^15^ but this may not be due to non-synonymous changes in the operon^19^. An alternative but less common mechanism of colistin resistance is through mutations in the *lpx* lipid A biosynthesis genes^20^. Mutations within *lpxACD* result in total loss of lipid A production and therefore LPS^20^. Although the cells remain viable there was an increased susceptibility to other antimicrobials, surprisingly clinical colistin resistant isolates with these mutations have been isolated^20^. Recently a TnSeq analysis of colistin resistance identified over 30 genes involved in inducible colistin resistance^21^. The study also identified that a *lpsB* knock-out, involved in LPS core biosysnthesis, increased sensitivity to colistin^21^. Phenotypically these mutations also have been shown to increase susceptibility to other antibiotics, in particular aminogylocides as well as phenol based biocides^18^. Analysis of the effect of these mutations on virulence in the *Galleria mellonaria* model has shown that, in some cases, virulence was not reduced^18^. Alarmingly a recent Chinese study identified plasmid borne phosphoethanolamine transferase enzyme (*mcr-1*) that conferred colistin resistance to *E. coli*^22^. The *mcr-1* gene has subsequently been identified in other countries including Vietnam^23^ where it was found with other AMR genes within pigs destined for food. Currently this has not been identified in *A. baumannii* but this this would appear to be just a matter of time.

Here we aimed to explore potential mechanisms of colistin resistance in *A. baumannii* by combining laboratory-induced resistance with high-throughput genome sequencing to identify subtle genomic changes resulting in reduced sensitivity to colistin. Previously reported colistin resistance mechanisms as well as several novel genetic targets were identified. *A. baumannii* can rapidly develop colistin resistance through multiple mechanisms that would be difficult to monitor through simple molecular diagnostic tests and has important implications for the future treatment of this rapidly emerging pathogen.

## Results

### Whole genome analysis of four Vietnamese A. baumannii clinical isolates

To elucidate how *de novo* mutations play a role in colistin resistance in *A. baumannii* we selected four colistin susceptible clinical isolates from patients with culture confirmed VAP in the Hospital for Tropical Diseases in Ho Chi Minh City between 2009 and 2011^9^ (Table 1). Additionally, the fully sequenced colistin susceptible *A. baumannii* ATCC19606 (CGN-35) was used^24^. Antimicrobial susceptibility testing showed that BAL266 was susceptible to the majority of antimicrobials tested (with the exception of ampicillin), while BAL062 and BAL242 and BAL255 were resistant to the majority of antimicrobials tested (Table 1). In addition, BAL255 was found to contain (by PCR amplification prior to genome sequencing) the *bla*_NDM-1_ gene, facilitating high-level resistance to carbapenems and third generation cephalosporins.

Draft genome sequences were produced, assembled and annotated for all five *A. baumannii* isolates. A multi-locus sequence type (MLST) BLAST database was used to derive the isolates sequence type (ST) (Table 2). BAL062 was identified to be an ST136 strain, an ST that was first identified in China. Furthermore, BAL242 belonged to ST493 (first identified in Vietnam), BAL255 belonged to ST355 (first identified in USA) and BAL266 was a unique ST (ST585), with no comparable isolates in the *A. baumannii* MLST database. Lastly ATCC19606 was identified to be a likely recombinant and contained a novel combination of previously identified alleles that contradicted the *A. baumannii* MLST database (Table 2). The *bla*_OXA-51_-like β-lactamase gene was identified by PCR^25^ in all four Vietnamese *A. baumannii* strains (Table 1). However, genome sequences further refined the presence of these genes to the *bla*_OXA-51_-like loci; *bla*_OXA-66_, *bla*_OXA-70_, *bla*_OXA-78_ and *bla*_OXA-98_ (Supplementary Table 1). The *A. baumannii* strain MDR-TJ^26^, a multidrug resistant (MDR) strain isolated in Tianjin, China was found to harbour a *bla*_OXA-23_ on a plasmid. During analysis of BAL062 and BAL242, the *bla*_OXA-23_ gene was identified but not the MDR-TJ-like plasmid, suggesting a chromosomal location, or an alternative resistance plasmid structure, for this gene in these organisms.

*A. baumannii* MDR-TJ was used as a reference sequence to identify the compendium *A. baumannii* of antimicrobial resistance (AMR) genes harboured by the Vietnamese strains (Supplementary Table 1)^26^,^27^. Analysis of the four Vietnamese *A. baumannii* strains and ATCC19606 found that many previously described *A. baumannii* AMR genes were highly conserved within these genetically variable isolates (Supplementary Table 1). Notably, the *gyrA* mutation S81L in *A. baumannii* MDR-TJ^27^, which induces reduced susceptibility against the fluoroquinolones, was additionally present in BAL062, BAL242 and BAL255. Furthermore, in comparison to *A. baumannii* MDR-TJ strain BAL255 contained an additional *gyrA* mutation, R442L, which may have sequential effect on increasing the Minimum Inhibitory Concentration (MIC) against fluoroquinolones.

### Analysis of mutations in colistin resistant progeny strains

A series of longitudinal (five days) sub-culturing experiments was performed to induce and increase the MIC against colistin in the four selected Vietnamese clinical *A. baumannii* isolates and ATCC19606. During these experiments the organisms were independently exposed to increasing concentrations of colistin. Firstly, the MIC against colistin was determined for all strains (Day 1 in Table 3) and then they were independently cultured on Mueller-Hinton agar supplemented with several differing concentrations of colistin (0.5, 1, 2, 4, 8, 16, 32 and 64 μg/μl). More than one colony growing on the highest concentration of colistin was selected and their MICs against colistin were measured. These strains were then independently sub-cultured on a higher range of colistin concentrations and again the organisms growing on the highest concentration of colistin were selected. After five days of sub-culture on increasing concentrations of colistin we had produced more than 30 strains that had MICs against colistin of >256 μg/μl. Maximal daily MIC for each strain is shown in Table 3 and we were able to generate colistin resistant mutants (according to the current colistin resistant breakpoint as determined by British Society for Antimicrobial Chemotherapy (≥8 μg/μl)) within three days for all five isolates.

Fifteen progeny colonies from the sub-culturing experiments, with a range of MICs against colistin, were fully genome sequenced (Table 4, Supplementary Table 2). Sequences from progeny colonies were aligned to the draft respective parent strains genome sequences to identify the induced genetic variation that may be responsible for the observed increase in MIC against colistin (Table 4). Over the period of five days of subculture from within the selected organisms we observed 17 different non-synonymous mutations and a single synonymous intergenic mutation in comparison to the parent isolates (Table 4).

We first compared the *pmrAB* genes, which are known hotspots for mutations facilitating elevated MICs against colistin. On analysis of *pmrAB* we found that this region exhibited 100% DNA sequence similarity in all strains prior to experimentation. Notably, after five days of subculture we observed no mutations in *pmrA* compared to the parent strains. Similarly, the DNA sequence of *pmrB* was identical in all parental isolates with the exceptions of a non-synonymous H440N substitution in ATCC19606 and an A444V inducing substitution in BAL062 and BAL242 in comparison to the *A. baumannii* MRD-TJ. These mutations were observed at the extreme 3′ end of the coding sequence and were predicted not to result in a change of susceptibility against colistin. Indeed the entire collection of parent isolates had an MIC of 1.5 μg/ml or below. In the resulting progeny with elevated MICs to colistin, the only strains to harbour non-synonymous mutations in *pmrB* originated from the parent BAL266 (Table 4, Supplementary Table 2). A pfam domain search of PmrB indicated that two histidine kinase domains are present between residues 218–279 and 326–436. Markedly, only PmrB from BAL266-Day3 contained an amino acid change (E229D) within these motifs, yet isolates with higher MIC values contained non-motif located PmrB mutations, for example R134C (BAL266-Day4), Y194S (BAL266-Day5) and G315S (BAL266-Day4 and BAL266-Day5) (Table 4).

The gene *pmrC* was present in all parental strains, however an accurate assembly of the gene sequence was confounded by the presence of a highly similar *pmrC*-compensating gene, *eptA*. Gene *eptA* encodes a phosphoethanolamine transferase, which is not proximate to the *pmrAB* locus^28^. Gene *eptA* is present in some strains and can occur in more than one copy, which would further complicate genome assemblies, and may induce a phenotypic effect on colistin susceptibility. BAL062, BAL266 and ATCC19606 had a single copy of *eptA*. BAL242, due to read depth and single nucleotide polymorphisms (SNPs) appears to have at least two copies despite a single copy in the assembly. BAL255, despite the lack of *eptA* in the assembly may have a copy as the sequence reads map against the *A. baumannii* MDR-TJ (CP003500) *eptA*.

The genes *lpxA* and *lpxD,* which encode acyltransferases for lipid A biosynthesis, are located in the *A. baumannii* genome, with only a single gene (*fabZ*) separating them; conversely, *lpxB* and *lpxC*, which are again involved in lipid A biosynthesis, are encoded in separate chromosomal regions. For example, in the completed *A. baumannii* 1656-2 genome^29^, using the systematic gene numbering, *lpxA, lpxD, lpxB* and *lpxC* equate to coding sequence number 2552, 2554, 2156 and 3579 respectively. Within the collection of colistin resistant progeny we were able to identify mutations in *lpxACD* but not *lpxB* (Table 4). Two BAL242 progeny colistin resistant isolates (BAL242-Day3 and BAL242-Day5) had an identical SNP in *lpxA* that resulted in the formation of a premature stop codon, truncating the protein at 325 amino acid residues. BAL255-Day3 had a non-synonymous A141T mutation in *lpxA*, falling within one of three predicted transferase hexapeptide repeats and a UDP N-acetylglucosamine O-acyltransferase (Domain 2). In *lpxD* three independent mutations resulted in abrogated LpxD at residue 325 (BAL062-Day5, BAL242-Day2 and BAL266-Day3); a loss of an adenine resulting as a T319R (BAL062-Day5 and BAL242-Day2) and a caa/Q to taa/stop mutation (BAL266-Day3). LpxC mutations occurred in BAL266-Day3 with a P30L substitution but a frameshift in ATCC19606-Day5 resulted in a truncated protein of 127aa (Table 4).

Lastly, additional analysis of the sequence data identified a number of other, not previously described, mutations that were associated with a colistin resistance phenotype: i) *zndP* and *pldA* are co-located in a putative operon; BAL255-Day4 had a frameshift in *zndP* which would result in a highly truncated protein (only 165/992 aa) and BAL266-Day3 (209/383) had mutation in *pldA,* ii) *vacJ* mutations occurred as frameshifts in both BAL242-Day5 and ATCC19606-Day3 resulting in truncated proteins of 257 and 135 aa respectively, iii) a N138S in *pheS* is involved in phenylalanyl-tRNA synthetase the mutation, iv) two hypothetical proteins also demonstrated significant truncation due to frameshifts; BAL255-Day5 Ab09_2943 and *ttg2C* (Table 4). Ab09_2943, an orthologue to *A. baumannii* 307-0294 CDS ABBFA_000135^30^ and conserved in all five strains. Ab09_2943 is a conserved hypothetical protein with similarity to a signal peptide protein of the putative MetA-pathway of phenol degradation^30^. *ttg2C* was similar to a toluene tolerance efflux ABC transporter periplasmic substrate-binding protein.

## Discussion

The rise of *A. baumannii* in Vietnam has been accompanied by the rapid emergence of antimicrobial resistance in this problematic environmental bacteria resulting in colistin being a last-resort antibiotic^9^. As resistance to colistin is primarily caused by spontaneous mutation as opposed to the acquisition of horizontally transferred resistance genes, we investigated which mutations could be induced under laboratory conditions in four MDR isolates of *A. baumannii*. We used whole genome sequencing to investigate MLST, antibiotic resistance gene content and mutations associated with colistin resistance.

On extracting the MLST alleles from the assembled genomes and inferring sequence type we found that each of the Vietnamese clinical isolates had a differing ST. ATCC19606, originally isolated in 1911 in Delft, The Netherlands, was present in the pubMLST database as an ST26 and used a control for the study (http://pubmlst.org/abaumannii/). However, analysis of ATCC19606 genome identified this strain as a novel/undocumented ST consisting of a new combination of known alleles. An independent shotgun assembly of the ATCC19606 genome was available at NCBI (http://www.ncbi.nlm.nih.gov/Traces/wgs/?val=ACQB01) and analysis of this genome assembly also produced an identical allele combination. The ATCC19606 ST and ST26 only share two alleles (*gltA* and *gyrB*) but *gdhB* differs by four SNPs, and *recA*, *cpn60*, *gpi* and *rpoD* all by one SNP. It is clear that the alleles are closely related so this discrepancy may be due to changes induced during long-term storage and sub-culture of this strain since it entered the ATCC.

Analysis of the Vietnamese clinical strains showed that many of the antimicrobial resistance associated genes were conserved between strains and found to be additionally present in the Chinese MDR-TJ strain^27^. BAL266 was phenotypically sensitive to all antimicrobials tested and was highly similar to the 1911 ATCC19606 AMR genotyping profile. BAL242 contained all MDR-TJ AMR genes except *aacC1* gentamycin resistance although it was BAL255 that had the *bla*_*ndm-1*_ genotype, giving high level resistance to carbapenems and third generation cephalosporins.

The association of mutations in the putative two component regulatory system PmrAB with resistance to colistin in *A. baumannii* was first described in 2009^15^. No mutations were observed within PmrA (Table 4), a response regulator which auto-regulates PmrABC expression; the same was observed for PmrC. The gene *pmrB*, a sensor kinase, has previously been shown to contain non-synonymous SNPs, frameshifts and deletions^15^,^28^. This study has identified further mutations associated with resistance against colistin in PmrB (Table 4). This suggests that variation within the sensor transcriptional regulator is the common mechanism for colistin resistance by altering gene expression. The loss of lipid A, and ultimately LPS, through mutations in the *lpxACD* genes in laboratory culture results in lower fitness and a low virulence potential of the organism^31^, however a few clinical isolates have been shown to possess *lpxACD* mutations^20^. All the *lpxACD* mutations generated in this study, with the exception of an LpxC P30L, have not been previously induced^20^.

Supplementary to mutations in the *pmrAB* and *lpxACD* genes, which have previously been demonstrated to have a role in colistin resistance, mutations in several other genes were additionally observed. The identified genes (e.g. *vacJ* and *pldA*) have not previously been implicated in having a role in changing antimicrobial susceptibility, however we note that the majority of the target genes are associated with the outer membrane, the target of polymyxins. In some of the sequenced strains an increase in the MIC against colistin can only be explained by these additional mutations. For example, both BAL 242-Day3 and BAL 242-Day5 had a premature stop codon in *lpxA* stop, but differed considerably in MIC (48 μg/μl and192 μg/μl, respectively). The only additional difference between these strains was frameshift in *vacJ* in BAL 242-Day5 (Table 4). Gene *vacJ* is a highly conserved and widely distributed outer membrane lipoprotein, important for virulence in a number of Gram-negative pathogens^32^,^33^. VacJ is frequently associated with Vps (Mla) to form the Vps-VacJ ABC transporter system, which is responsible for maintaining outer-membrane asymmetry (LPS at the outer leaflet and phospholipids at the inner leaflet)^34^. Bacteria accumulate phospholipids in the outer leaflet, for example after exposure to chemical treatments, disrupting LPS organization and increasing sensitivity to toxic compounds^34^. VacJ was found to be upregulated in a putative outer membrane protein mutant of *S. enterica* serovar Typhimurium that resulted in resistance against ceftriaxone^35^. Mutations within the *vps-vacJ* transporter genes result in increased sensitivity to Sodium dodecyl sulfate (SDS) in both *E. coli*^34^ and *Shigella flexneri*^36^. A spontaneous *pldA* mutation, resulting in increased transcription, restored the SDS insensitivity^34^. PldA has been suggested to remove phospholipids in the outer leaflet of the outer membrane, maintaining asymmetry, an activity which has been shown to increase in bacteria with destabilised outer membranes^37^. In this study two *vacJ* mutants and a *pldA* mutation were identified in colistin resistant progeny, predicting that outer asymmetry maintenance is related to colistin susceptibility. Interestingly, *pldA* is downstream of *zndP*, in which another putative colistin resistance associated mutation was identified. ZndP is a putative zinc-dependant peptidase, and when linked to PldA, may have a role in outer membrane processing. Ttg2C is a putative solvent/toluene tolerance efflux ABC transporter periplasmic substrate-binding protein which has been shown to be upregulated in *Pseudomonas putida* KT2440 in response to phenol exposure^38^. Phenol is an organic solvent that solubilises the cell wall, the same mode of action as colistin, and hence genes involved in phenol resistance may confer colistin resistance. Interestingly this study also identified VacJ amongst many upregulated proteins^38^. These additional genes were not identified as differentially expressed under NaCl induced colistin resistance^21^.

Here we sought to induce and identify mutations associated with colistin resistance in *A. baumannii*. Taking several antimicrobial resistant, but colistin susceptible Vietnamese clinical isolates of *A. baumannii*, we induced rapid high-level resistance to colistin. In performing whole genome sequencing of colistin resistant *A. baumannii* strains we aimed to take a holistic approach to the genetics behind resistance. Previous studies have focused specifically on the PmrAB and LpxACD (for example^18^) but other studies have demonstrated other genes are involved (for example *lpsB*^21^). We have identified mutations within genes that can be hypothesised to play a role due to linkage to the other membrane.

In conclusion, colistin resistant *A. baumannii* can be generated rapidly in a laboratory. Heightened surveillance in clinical laboratories and across international networks should be perform to detect these organisms rapidly when they arise and prevent their spread. Our work worryingly shows that colistin, a drug of last resort, may rapidly become ineffective, having serious implications for future treatment of infections caused by *A. baumannii* and other hospital acquired pathogens.

## Methods

### Bacterial identification and antimicrobial susceptibility testing

The reference *A. baumannii* Bouvet and Grimont strain from 1911 was purchased from ATCC (ATCC19606). Clinical bacterial strains were isolated from tracheal aspirates (TA) specimens taken from patients with suspected VAP in the adult ICU of the Hospital of Tropical Diseases in Ho Chi Minh City, Vietnam from January 2011 to June 2012 (Table 1). *A. baumannii* were identified using API20NE and confirmed by using a previously described PCR method to detect *bla*_*OXA-51*_, which is intrinsic to the species^39^. All *A. baumannii* isolates were stored in glycerol at −80 °C until further analysis.

Antimicrobial susceptibilities were determined at the time of isolation by the modified Bauer-Kirby disk diffusion method, as recommended by the CLSI guidelines (CLSI. 2011/2. Performance Standards For Antimicrobial Susceptibility Testing; Twenty-First Informational Supplement, CLSI document M100-S21, vol. 31, Clinical and Laboratory Standards Institute) and included Ampicillin (AMP-10 μg), piperacillin/tazobactam (TZP-100/10 μg), imipenem (IMP-10 μg), amikacin (AMK-30 μg), ofloxacin (OFX-5 μg), ceftazidime (CAZ-30 μg), ceftriaxone (CRO-30 μg), co-trimoxazole (SXT-1.25/23.75 μg), cefepime (FEP-30 μg), gentamycin (GEN-10 μg) and colistin (CST-10 μg). Mueller-Hinton agar and antimicrobial discs were purchased from Unipath, Basingstoke, United Kingdom. For colistin (CST), imipenem (IMP), ceftazidime (CAZ), and ceftriaxone (CRO), minimum inhibitory concentrations (MICs) were determined by E-test based on manufacturer’s recommendations (AB Biodisk, Sweden). The results were interpreted as resistant or sensitive according to current CLSI guidelines. *Escherichia coli* ATCC 25922 was used as the control for these assays.

### Induction of resistance

All clinical isolates were recovered from glycerol stock on Mueller-Hinton (MH) plates and colonies of each organism were suspended into 100 μl of sterile phosphate buffered saline (PBS). To induce resistance to colistin 3 μl of each suspension was inoculated on MH plates supplemented with differing concentrations of colistin (0.5, 1, 2, 4, 8, 16, 32 and 64 μg/μl) for five consecutive days or until the highest MIC to colistin was reached. The colistin concentration used in the inducement experiment varied among *A. baumannii* strains according to their corresponding MIC to colistin, which had been determined by E-test strip, and ranged from 1 x MIC to 8 x MIC for each strain. Colonies of each strain that were able to grow on MH plates with the highest colistin concentration for each day were inoculated in glycerol and stored at −80 °C until DNA extraction.

### Whole genome sequencing and analysis

Genomic DNA was extracted using the Wizard Genomic DNA Extraction Kit (Promega, Fitchburg, WI). The quality and concentration of the DNA were assessed using a Nanodrop bioanalyser spectrophotometer (Thermo Scientific, Delaware, USA). All isolates were sequenced on an Illumina MiSeq using the Nextera XT as per the manufacturers’ protocols. All samples were multiplexed and sequenced on a single MiSeq 2 × 151 bp run (EBI: PRJEB10709). Demultiplexed FASTQ files were quality controlled using Trimmomatic-0.30 [LEADING:3 TRAILING:3 SLIDINGWINDOW:4:20 MINLEN:36]^40^. Draft genomes were *de novo* assembled using VelvetOptimiser-2.2.5 (Victorian Bioinformatics Consortium, Australia) and Velvet 1.2.09^41^. Contigs from the sensitive parental strains were ordered by Abacas 1.3.1^42^ using *A. baumannii* MDR-TJ (CP003500) as a reference. Contigs breaks were joined using IMAGE^43^. Derived progeny draft assembly contigs were ordered to the parental strain using Abacas. Genomes were annotated using Prokka and an custom Acinetobacter reference library^44^. Laboratory induced SNPs were identified by mapping progeny sequencing reads against the parental isolate using BWA^45^ and SAMtools^46^ [varFilter.pl]. All SNPs were manually checked. MLST was determined using BLAST^47^ and a custom allele database.

## Additional Information

**How to cite this article**: Thi Khanh Nhu, N. *et al.* The induction and identification of novel Colistin resistance mutations in *Acinetobacter baumannii* and their implications. *Sci. Rep.* **6**, 28291; doi: 10.1038/srep28291 (2016).

## Supplementary Material

Supplementary Information

## Figures and Tables

**Table 1:** Antimicrobial susceptibility profiles of the parent *A. baumannii* strains used in this study.

Isolate	Date	AMP	TZP	IMP	AMK	OFX	CAZ	CRO	SXT	FEP	GEN	CST	NDM -1	OXA -51	OXA -23	OXA -24	OXA -58
BAL 062	Apr-09	R	R	R	R	R	R	R	R	R	R	S	–	+	+	–	–
BAL 242	Jan-11	R	R	R	R	R	R	R	R	R	R	S	–	+	+	–	–
BAL 255	Feb-11	R	S	R	R	R	R	R	R	R	R	S	+	+	–	–	+
BAL 266	Feb-11	R	S	S	S	S	S	S	S	S	S	S	–	+	–	–	–

AMP; ampicillin, TZP; piperacillin/tazobactam, IMP; imipenem, AMK; amikacin, OFX; Ofloxacin, CAZ; ceftazidime, CRO; ceftriaxone, SXT; co-trimoxazole, FEP; cefepime, GEN; gentamycin, CST; colistin.

**Table 2:** Genome characteristics of the sequenced *A. baumannii* isolates in this study.

Isolate	Bp (GC%)	Coverage	CDSs	*gltA*	*gyrB*	*gdhB*	*recA*	*cpn60*	*gpi*	*rpoD*	ST	Origin
BAL 062	4,028,838 (38.99)	41.2x	3,960	1	3	3	2	2	16	3	ST136	This study
BAL 242	3,912,277 (38.91)	33.6x	3,786	1	15	3	2	2	106	3	ST493	This study
BAL 255	3,953,867 (39.02)	64.5x	3,752	1	81	11	48	18	114	43	ST355	This study
BAL 266	3,774,702 (38.86)	43.8x	3,645	1	15	13	12	4	163	2	ST585	This study
ATCC19606	3,974,237 (39.06)	49.6x	3,894	1	10	8	6	1	110	14	New	[34, 35]

Bp = approximate genome size in base pairs, CDSs = Prokka predicted number of coding sequences, ST = Multilocus sequence typing (MLST) sequence type. *gltA*, *gyrB*, *gdhB*, *recA*, *cpn60*, *gpi* and *rpoD* are MLST scheme genes, unique alleles given unique number and combination of seven alleles define a strain ST.

**Table 3:** *A. baumannii* susceptibilities to Colistin *in vitro.*

Isolate	Minimum inhibitory concentration against colistin μg/ul				
	Day 1	Day 2	Day 3	Day 4	Day 5
BAL 062	0.5	0.75	>256	>256	>256
BAL 242	0.5	32	48	192	192
BAL 255	0.5	0.75	48	128	>256
BAL 266	1.5	6	64	96	128
ATCC19606	0.5	16	>256	>256	>256

**Table 4:** Summary of mutations in colistin resistant progeny.

Strain	MIC to Colistin (ug/ml)	LpxA (262)	LpxC (300)	LpxD (356)	PmrB (444)	PheS (326)	ZndP (992)	PldA (383)	VacJ (299)	135 (255)	Ttg2C (226)
BAL062	0.5										
BAL062-Day2	0.75										
BAL062-Day5	>256			T319R (325)†							N104M (105)†
BAL242	0.5										
BAL242-Day2	32			T319R (325)†							
BAL242-Day3	48	STOP (229)									
BAL242-Day5	192	STOP (229)							Q249T (257)†		
BAL255	0.5										
BAL255-Day2	0.75										
BAL255-Day3	48	A141T		H242L*							
BAL255-Day4	128			H242L*			F149L (165)†				
BAL255-Day5	>256			H242L*						W179C (186)†	
BAL266	1.5										
BAL266-Day2	6										
BAL266-Day3	16		P30L					T200T (209)†			
BAL266-Day4	96				R134C, G315S						
BAL266-Day5	128				Y194S, G315S	N138S					
ATCC19606	0.5										
ATCC19606-Day3	>256								R166N (135)†		
ATCC19606-Day5	>256		Y111C (127)†								

135 = *A. baumannii* AB307-0294 CDS ABBFA_000135 homologue annotated as ‘hypothetical protein.

^*^indicates a synonymous change gca- >gcg (A->A) was also present.

^†^Denotes a frameshift due to a nucleotide deletion/insertion resulting in a truncated protein, (xx) indicates the size of full/truncated protein in amino acids, STOP indicates a C to T SNP that generates a TAA stop codon.
