# Supplementary material for: The induction and identification of novel Colistin resistance mutations in Acinetobacter baumannii and their implications
Source: Sci Rep. 2016 Jun 22;6:28291. doi: 10.1038/srep28291 (PMC4916428; doi:10.1038/srep28291)
Supplement: Supplementary Information [file srep28291-s1.pdf]

The induction and identification of novel Colistin resistance mutations in *Acinetobacter baumannii* and their implications.

Nguyen Thi Khanh Nhu [1,2], David W. Riordan [3], Tran Do Hoang Nhu [1], Duy Pham Thanh [1],

Guy Thwaites [1,4], Nguyen Phu Huong Lan [5], Brendan W. Wren [3], Stephen Baker [1,3,4] and Richard A Stabler [3]*

Supplementary table 1. Identification of antimicrobial resistance genes by comparison to *A. baumannii* AMR-TJ

Category	Function	Gene	MDR-TJ§	BAL 062	BAL 242	BAL 255	BAL 266	ATCC 19606
Efflux pumps	RND family efflux pump	*adeT*	ABTJ_00008	Yes	Yes	Yes	Yes	Yes
Efflux pumps	RND family efflux pump	*adeT*	ABTJ_00009	Yes	Yes	Yes	Yes	Yes
Efflux pumps	RND family efflux pump	*adeT*	ABTJ_00010	Yes	Yes	Yes	Yes	Yes
Efflux pumps	MATE family efflux pump	*abeM*	ABTJ_00064	Yes	Yes	Yes	Yes	Yes
Efflux pumps	MATE family efflux pump	*abeM*	ABTJ_00108	Yes	Yes	Yes	Yes	Yes
Heavy metals	Cobalt–zinc–cadmium resistance	*czcB/A*	ABTJ_00275	Yes	Yes	No	No	No
Heavy metals	Cobalt–zinc–cadmium resistance	*czcB/A*	ABTJ_00276	Yes	Yes	No	No	No
Efflux pumps	RND family efflux pump	*adeT*	ABTJ_00399	Yes	Yes	Yes	Yes	Yes
β-Lactamases	Putative sulphide–quinone reductase	*blh*	ABTJ_00521	Yes	Yes	Yes	Yes	Yes
Efflux pumps	SMR family efflux pump	*qacEΔ1*	ABTJ_00612	Yes	Yes	Yes	Yes	Yes
Efflux pumps	RND family efflux pump	*adeIJK*	ABTJ_00725	Yes	Yes	Yes	Yes	Yes
Efflux pumps	RND family efflux pump	*adeIJK*	ABTJ_00726	Yes	Yes	Yes	Yes	Yes
Efflux pumps	RND family efflux pump	*adeIJK*	ABTJ_00727	Yes	Yes	Yes	Yes	Yes
Chloramphenicol	Chloramphenicol acetyltransferase	*catB2*	ABTJ_00778	Yes	Yes	Yes	Yes	Yes
Sulphonamides	Dihydropteroate synthase type 1	*sul1*	ABTJ_00787	Yes	Yes	Yes	Yes	Yes
β-Lactamases	Class C β-lactamase	*ampC*†	ABTJ_01149	Yes	Yes	Yes	Yes	Yes
Efflux pumps	SMR family efflux pump	*qacEΔ1*	ABTJ_01221	Yes	Yes	Yes	Yes	Yes
Aminoglycosides	Aminoglycoside 6′-acetyltransferase	*aacA4*	ABTJ_01325	No	Yes	No	No	No
Chloramphenicol	Chloramphenicol acetyltransferase	*cat*	ABTJ_01326	No	Yes	No	No	No
Aminoglycosides	Streptomycin 3″-adenylyltransferase	*aadA1**	ABTJ_01327	No	Yes	No	No	No
Sulphonamides	Dihydropteroate synthase type 1	*sul1**	ABTJ_01329	No	Yes	No	No	No
Aminoglycosides	Ribosomal RNA methyltransferase	*arm*	ABTJ_01332	No	Yes	No	No	No
β-Lactamases	Class C β-lactamase	*ampC*†	ABTJ_01490	Yes	Yes	Yes	Yes	Yes
β-Lactamases	Metallo-β-lactamase superfamily	??	ABTJ_01722	Yes	Yes	Yes	Yes	Yes
Aminoglycosides	Aminoglycoside 6′-acetyltransferase	*aacA4*	ABTJ_01726	Yes	Yes	Yes	Yes	Yes
Efflux pumps	RND family efflux pump	*adeT*	ABTJ_01876	Yes	Yes	Yes	Yes	Yes
Efflux pumps	RND family efflux pump	*adeABC*	ABTJ_01879	Yes	Yes	Yes	Yes	Yes
Efflux pumps	RND family efflux pump	*adeABC*	ABTJ_01880	Yes	Yes	Yes	Yes	Yes
Efflux pumps	RND family efflux pump	*adeABC*	ABTJ_01881	Yes	Yes	Yes	Yes	No

β-Lactamases	Class C β-lactamase	*ampC*†	ABTJ_01965	Yes	Yes	Yes	Yes	Yes
β-Lactamases	Class D β-lactamase	*blaOXA-66*	ABTJ_02147	Yes	Yes	Yes‡	Yes¶	Yes#
Heavy metals	Arsenical resistance protein	*acr3*	ABTJ_02215	Yes	Yes	Yes	Yes	Yes
β-Lactamases	Putative class A β-lactamase	??	ABTJ_02449	Yes	Yes	Yes	Yes	Yes
Aminoglycosides	Gentamicin 3′-acetyltransferase	*aacC1*	ABTJ_02576	Yes	No	No	No	No
Aminoglycosides	Streptomycin 3″-adenylyltransferase	*aadA1**	ABTJ_02579	Yes	Yes	Yes	No	No
Sulphonamides	Dihydropteroate synthase type 1	*sul1**	ABTJ_02581	Yes	Yes	Yes	No	No
Heavy metals	Cobalt–zinc–cadmium resistance	*czcD*	ABTJ_03277	Yes	Yes	Yes	Yes	Yes
Efflux pumps	MATE family efflux pump	*abeM*	ABTJ_03383	Yes	Yes	Yes	Yes	Yes
β-Lactamases	Class C β-lactamase	*ampC*	ABTJ_03416	Yes	Yes	Yes	Yes	Yes
Aminoglycosides	Aminoglycoside 3′-phosphotransferase	*strA*	ABTJ_03558	Yes	Yes	Yes	Yes	Yes
Aminoglycosides	Aminoglycoside 6′-phosphotransferase	*strB*	ABTJ_03559	Yes	Yes	Yes	Yes	Yes
Efflux pumps	MFS family efflux pump	*tetA(B)*	ABTJ_03564	Yes	Yes	Yes	Yes	Yes
Sulphonamides	Dihydropteroate synthase type 2	*sul2*	ABTJ_03576	No	Yes	Yes	No	Yes
β-Lactamases	Metallo-β-lactamase superfamily	???	ABTJ_03808	Yes	Yes	Yes	Yes	Yes
Heavy metals	Cobalt–zinc–cadmium resistance	*czcD*	ABTJ_03832	Yes	Yes	Yes	Yes	Yes
β-Lactamases	Class D β-lactamase	*blaOXA-23*	ABTJ_p0085	Yes	Yes	No	No	No

Whole genome sequencing reads were mapped against the drug resistance reference strain *A. baumannii* MDR-TJ to identify conserved genotypes. RND = resistance-nodulation-division, MATE = multidrug and toxic compound extrusion, SMR = small multidrug resistance, MFS = major facilitator superfamily, *§* = Systematic gene numbering from *A. baumannii* MDR-TJ (EMBL: CP003500), † *ampC* was present in triplicate in all genomes, * *aadA1* and *sul1* are duplicated in *A. baumannii* MDR-TJ therefore for Ab01/BAL062 and Ab09/BAL255, which contain a single copy, the flanking genes/MGE were used to identify the homologous copies. *bla*OXA-66, belongs to the diagnostic blaOXA-51-like group; *‡* = *bla*OXA-51-like match (*bla*OXA-70), ¶ = *bla*OXA-51-like match (*bla*OXA-68), # = *bla*OXA-51-like match (*bla*OXA-98).

Supplementary table 2. Identified mutations in colistin resistant progeny.

Strain	Av read depth	MIC to Colistin (ug/ml)	Gene	Wt	Mt	bp	Cov
BAL062-Day2	35	0.75					
BAL062-Day5	32	>256	LpxD	gaaaaaaa	gaaaaaa	3627100	20
			Ttg2C	caaaaaaa	caaaaaa	2641771	18
BAL242-Day2	31	32	LpxD	gaaaaaaa	gaaaaaa	3354479	37
BAL242-Day3	71	48	LpxA	C	T	3355778	60
BAL242-Day5	39	192	LpxA	C	T	3355778	26
			VacJ	ta	tAa	1169000	55
BAL255-Day2	31	0.75					
BAL255-Day3	44	48	LpxA	G	A	809212	70
			LpxD	A	T	807957	54
BAL255-Day4	51	128	LpxD	A	T	807957	44
			ZndP	t	ta	972601	46
BAL255-Day5	40	>256	LpxD	A	T	807957	32
			135	gcagtcactgcccagt	gcagt	3426042	18
BAL266-Day2	38	6					
BAL266-Day3	53	16	LpxC	C	T	2526124	56
			PldA	accc	aCccc	2926974	44
BAL266-Day4	58	96	PmrB	C	T	118932	49
			PmrB	G	A	119475	61
BAL266-Day5	33	128	PmrB	A	C	119113	28
			PmrB	G	A	119475	23
			PheS	T	C	3525158	52
ATCC19606-Day3	19	>256	VacJ	gaatata	gAATATaatata	3436514	15
ATCC19606-Day5	15	>256	LpxC	catttatttattt	catttattt	454944	12

Strain = Parent and progeny designation, ARD = average read depth of mapped trimmed reads across the parent genome, Gene = target gene annotation (135 = *A. baumannii* AB307-0294 CDS ABBFA_000135 homologue), Wt = allele in sensitive parent, Mt = mutant in resistant daughter isolate, bp = base of mutation according to parent genome, Cov = coverage at mutation site.
